# False-positive screening results in the Finnish prostate cancer screening trial

**DOI:** 10.1038/sj.bjc.6605512

**Published:** 2010-01-05

**Authors:** T P Kilpeläinen, T L J Tammela, L Määttänen, P Kujala, U-H Stenman, M Ala-Opas, T J Murtola, A Auvinen

**Affiliations:** 1 Department of Urology, University of Tampere and Tampere University Hospital, Box 2000, Tampere FIN-33521, Finland; 2 Tampere School of Public Health, University of Tampere, Tampere FIN-33014, Finland; 3 Finnish Cancer Registry, Pieni Roobertinkatu 9, Helsinki FIN-00130, Finland; 4 Department of Pathology, Tampere University Hospital, Box 2000, Tampere FIN-33521, Finland; 5 Department of Clinical Chemistry, Helsinki University Hospital, Box 700, Helsinki FIN-00029, Finland; 6 Department of Urology, Helsinki University Hospital, Box 580, Helsinki FIN-00029, Finland; 7 Department of Surgery, Central Finland Central Hospital, Jyväskylä FIN-40620, Finland

**Keywords:** mass screening, prostatic neoplasms, PSA, sensitivity and specificity, randomised controlled trials

## Abstract

**Background:**

There is evidence that prostate cancer (PC) screening with prostate-specific antigen (PSA) serum test decreases PC mortality, but screening has adverse effects, such as a high false-positive (FP) rate. We investigated the proportion of FPs in a population-based randomised screening trial in Finland.

**Methods:**

Finland is the largest centre in the European Randomized Study of Screening for Prostate Cancer. We have completed three screening rounds with a 4-year screening interval (mean follow-up time 9.2 years) using a PSA cutoff level of 4.0 ng ml^−1^; in addition, men with PSA 3.0–3.9 and a positive auxiliary test were referred. An FP result was defined as a positive screening result without cancer in biopsy within 1 year from the screening test.

**Results:**

The proportion of FP screening results varied from 3.3 to 12.1% per round. Of the screened men, 12.5% had at least one FP during three rounds. The risk of next-round PC following an FP result was 12.3–19.7 *vs* 1.4–3.7% following a screen-negative result (depending on the screening round), risk ratio 3.6–9.9. More than half of the men with one FP result had another one at a subsequent screen. Men with an FP result were 1.5 to 2.0 times more likely to not participate in subsequent rounds compared with men with a normal screening result (21.6–29.6 *vs* 14.0–16.7%).

**Conclusion:**

An FP result is a common adverse effect of PC screening and affects at least every eighth man screened repeatedly, even when using a relatively high cutoff level. False-positive men constitute a special group that receives unnecessary interventions but may harbour missed cancers. New strategies are needed for risk stratification in PC screening to minimise the proportion of FP men.

Prostate cancer (PC) is the most common cancer in most industrialised countries (Parkin *et al*, 2005). Its incidence increased steadily from the 1980s onwards, as the increased use of transurethral resection of the prostate for benign prostatic hyperplasia (BPH) resulted in more (incidental) PC diagnoses (Merrill *et al*, 1999). A steep rise in the incidence of PC was observed in the 1990s when the prostate-specific antigen (PSA) test as a diagnostic tool was adapted widely (Welch and Albertsen, 2009). Lately, the incidence of PC has been decreasing in many countries (Welch and Albertsen, 2009).

Screening for PC with PSA has become one of the most controversial public health issues. The two major screening trials in Europe and in the United States have provided inconsistent results concerning the mortality effects of PSA-based PC screening (Andriole *et al*, 2009; Schröder *et al*, 2009). Although it is essential to determine mortality and the quality-of-life effects of screening, it is also important to evaluate the sensitivity and specificity of the screening test to ensure best possible screening protocol, that is, maximise the benefits and minimise the harms of screening. The proportion of false-positive (FP) screening results indicates one aspect of adverse effects of screening, in addition to overdiagnosis and overtreatment.

The FP results are related to the specificity of the screening test. Specificity represents the ability of a test or test protocol to identify those free of the target disorder. Specificity is calculated as the ratio of the frequency of the true negative results (those with a negative test and without the target disease) to the sum of the frequencies of the true negatives and FPs (those with a positive test but free of the disease). Hence, the proportion of FP results is 1-specificity.

The FP screening results are common in PC screening, as PSA is an organ-specific but not a disease-specific marker (Stenman *et al*, 2000). It has been previously reported that ∼70% of men with elevated PSA do not have PC (Catalona *et al*, 1994; Schröder *et al*, 1998). The proportion of FP results is likely to increase with age, as prostatic diseases, such as chronic prostatitis and BPH, become more common (Koskimäki *et al*, 1998; Rhodes *et al*, 1999; Wright *et al*, 2002). The proportion of FP results has been estimated to be 7–8% (Lafata *et al*, 2004; Määttänen *et al*, 2007) per screen (with 1 year of follow-up after the test). In repeated screening, the cumulative proportion of FP results was recently estimated at 10.4% with four PSA tests and over 3 years of follow-up (Croswell *et al*, 2009).

The purpose of our study was to assess the proportion of FP results in a population-based randomised controlled trial in Finland during three screening rounds. We evaluated whether men with an FP result are at greater risk of decreased screening compliance, subsequent PC, or repeated FP result(s). We also investigated how many biopsies men with FP results undergo and whether the use of medication for BPH affects FP rates.

## Materials and methods

The Finnish Prostate Cancer Screening Study is the largest component of the European Randomized Study of Screening for Prostate Cancer (ERSPC), which is a multicentre randomised trial. The Finnish trial comprises 80 255 men born during 1929–1944 (aged 55, 59, 63 or 67 years at entry) and residing either in the Helsinki or Tampere metropolitan area. Men with a previous PC diagnosis were excluded. Subjects were identified from the Finnish Population Registry. A random sample of 8000 men was allocated to the screening arm annually during 1996–1999 and the remaining men formed the control group that received no interventions and was not contacted either. This analysis covered only the screened men.

The men in the screening arm were sent an invitation letter along with a brief overview of the trial, a questionnaire about urological symptoms, as well as a family history of PC, previous PSA tests and an informed consent form.

The men in the screening arm were invited to give a blood sample at a local cancer society clinic in Helsinki or Tampere. Men with PSA ⩾4 ng ml^−1^ were referred to a urological clinic for diagnostic examinations, including digital rectal examination (DRE), transrectal ultrasound and biopsy. Initially, a sextant biopsy was used, but this was increased to 10–12 biopsy cores in 2002. Men with a PSA level of 3.0–3.9 ng ml^−1^ were referred to an additional test, which was DRE during 1996–1998 and since 1999 a free/total PSA (F/T PSA) ratio with a cutoff point of 16%. Men with a suspicious DRE or F/T PSA ratio <16% were referred to diagnostic examinations similar to those with PSA ⩾4.0 ng ml^−1^.

All the laboratory analyses were carried out at the Department of Clinical Chemistry, Helsinki University Hospital. The serum concentrations of total PSA were analysed by both Hybritech (Beckman Coulter, Inc., Brea, CA, USA) Tandem-E and Wallac Delfia (Wallac, Turku, Finland) assays. The free/total PSA ratio was determined with the Wallac ProStatus (Wallac) free/total PSA assay.

The men in the screening arm were then re-invited in a similar manner 4 and 8 years after the first screen to the second and third screening rounds (though men older than 71 years of age were no longer invited because the core age group in the protocol was 55–69 years of age). The first screening round was carried out during 1996–1999, the second during 2000–2003 and the third during 2004–2007. The common closing date of follow-up was 31 December 2007 with a mean follow-up of 9.2 years. All the men in the screening arm were invited to each round regardless of their participation in the previous round(s). Men diagnosed with PC were not re-invited, and neither were men who had emigrated from the study area or had died. Information on vital status and place of residence was obtained from the Population Register Centre.

Diagnosis of PC was based on histopathological examination. A re-biopsy within 2 months was indicated if the primary histopathological diagnosis was prostatic intraepithelial neoplasia, atypical small acinar proliferation or unconfirmed suspicion of PC, or if the PSA level was ⩾10 ng ml^−1^. The decision of re-biopsying a patient after a negative biopsy was made by the attending physician, who did not always comply with the protocol of the screening trial. Therefore, some re-biopsies were performed with less strict criteria and some postponed further than protocol-defined time frames. The definition of an FP result was a positive screening result (based on both total PSA and either DRE or free/total PSA ratio) and consequent diagnostic work-up with no histopathological diagnosis of PC in the biopsy within 1 year from the PSA test. The men who had a positive screening result but did not undergo biopsy according to the screening protocol were not analysed in this study.

Data on cancers detected outside the screening protocol were obtained from the nationwide, population-based Finnish Cancer Registry, which has 99% coverage of all solid cancers diagnosed in Finland (Teppo *et al*, 1994). Information on cancer incidence as well as vital status was available until the end of 2007. Data on BPH medication (finasteride or alpha blockers) use at the time of screening (during 1996–2004) were obtained by linking the study population to the prescription drug database of the Social Insurance Institution of Finland (SII, http://www.kela.fi/). The SII is a governmental agency providing reimbursements to the Finnish citizens for the cost of drugs prescribed by physicians (with the exception of hospital in-patients).

The 95% confidence intervals (CIs) for risks and proportions were calculated on the basis of basic s.e. formulas. A generalised linear model for binomial distribution with a logarithmic link function was used to calculate risk ratios (RRs) and their 95% CIs. The events were FP screening results and diagnosis of PC, with RRs indicating relative frequencies of outcomes in the groups to be compared. Statistical analyses were performed using Stata 8.2 (StataCorp, College Station, TX, USA).

## Results

Out of 30 195 men in the screening arm, 23 771 (78.7%) participated in at least one screening round, and 10 327 men (52.1% of those invited to all rounds) participated in all the three rounds.

Altogether, 1611 cancers were detected by screening, of which 543 were in the first round with a detection rate (DR) of 2.6%, 613 (DR 3.3%) in the second and 455 (DR 3.6%) in the third. The overall risk for an FP result was 6.4% in the first round, 8.0% in the second and 7.8% in the third. The risk of an FP result varied from 3.3 to 12.1% per round, depending on the screening round and age (Table 1). When men of similar age at screening were compared, the proportion of FP results was lower at repeat screening compared with the first round.

Of the men with a screen-positive result in the first round, 67.3% turned out to be FP and 27.5% were diagnosed with PC (5.2% of screen-positive men were not biopsied according to the protocol). In the second round, 64.6% of the screen-positive findings were FP and 26.6% PC, whereas in the third round 60.7% were FP and 27.7% PC. There was little variation by age (results not shown).

Of the 23 771 men who participated at least once during the three rounds, 12.5% (CI 12.1–12.9) had at least one FP result. The proportion of men with at least one FP result during the screening programme increased consistently with age from 9.0% in the youngest age cohort to 15.7% in the oldest age cohort (with only two screening rounds). Of the 10 327 men who participated in all three rounds, 1193 (11.6%, CI 10.9–12.2%) had at least one FP result. Of them, 1.2% (CI 1.0–1.4%) had an FP result in all three rounds, 2.8% (CI 2.5–3.1%) had it twice and 7.6% (CI 7.1–8.1%) had it once during the three rounds.

The risk of next-round PC diagnosis was 12.3–19.7% following an FP result *vs* 1.4–3.7% following a screen-negative result, RR 3.6–9.9 (age-stratified risks, RRs and their CI presented in Table 2). There were 128 men who had FP in the first round and were diagnosed with PC in the second round – 78.1% of the cancers were localised and of low grade (T1-2NxM0 and Gleason score <7), 13.3% were localised with Gleason score 7, and 8.6% were advanced (T3-4NxM0 or TxNxM1 or Gleason score ⩾8). In the men with a negative screening result in the first round and a screen-detected PC in the second round, the corresponding numbers were 77.5, 13.2 and 9.3%. Similarly, there were 77 cancers in the third round in those men who had FP in the second round; 57.1% were localised and of low grade (60.9% in the first round screen negatives), 29.9% were localised with Gleason score 7 (26.9%), and 13.0% were advanced (12.2%).

More than half of the men with one FP result had another in a subsequent round, whereas men with normal PSA levels had a 4.8–5.4% risk of next-round FP result (Table 2). Men with an FP result were 1.5–2.0 times more likely to not participate in subsequent rounds compared with men with normal screening results (21.6–29.6 *vs* 14.0–16.7%).

In the first round, moderately increased PSA concentration was associated with high probability of FP, whereas high PSA concentration (⩾10 ng ml^−1^) was associated with relatively high probability of PC (Table 3). Towards the third round, the probability of PC rose in the moderately increased PSA group and decreased in the high PSA group.

Information on the use of medication for BPH was available for 23 319 men. The number of men who had used medication for BPH (finasteride or alpha blockers or both) at first screen was 785 (3.8% of participants, mean age 62.5 years *vs* 60.1 years in men without BPH medication), at second screen 1870 (10.1% of participants, mean age 65.9 years *vs* 63.8 years) and at third screen 460 (14.5% of participants in the first year of the third round, mean age 67.1 years *vs* 66.3 years). The men with BPH medication had roughly twice the risk for FP result compared with men without BPH medication: risk for FP was 14.0 *vs* 6.1% in the first round, 13.4 *vs* 7.4% in the second round and 11.7 *vs* 8.5% in the third round. Age-adjusted first round RR was 1.9 (CI 1.5–2.2, *P*<0.001), second round RR was 1.6 (CI 1.4–1.8, *P*<0.001) and third round RR was 1.3 (CI 1.0–1.8, *P*-value=0.04). There was no increased risk for PC, as the risk in men with BPH medication *vs* men without BPH medication was 2.4 *vs* 2.6% in the first round (age-adjusted RR 0.8, CI 0.5–1.2, *P*-value=0.20), 3.8 *vs* 3.2% in the second round (age-adjusted RR 1.0, CI 0.8–1.3, *P*-value=0.99) and 7.2 *vs* 4.7% in the third round (age-adjusted RR 1.4, CI 1.0–2.0, *P*-value=0.08).

In the first round, men who were diagnosed with PC underwent on average 1.16 biopsies before diagnosis – that is, every sixth man underwent on average two biopsies. Men with an FP result had 1.30 biopsies in the follow-up time, that is, every third man received two biopsies. In the second round, men with PC had 1.13 biopsies and men with FP had 1.25 biopsies. These numbers decreased in the third round to 1.05 and 1.11, respectively. The maximum number of biopsies for an FP man was 7 (4 men) and for a man with PC 4 (1 man). Of the men with at least one FP result, 6.8% had three or more biopsies.

Of the 1331 men who had an FP result in the first round, 370 (27.8%) developed a PC in the following 8–11 years (128 were diagnosed at the second screen, 28 at the third screen and 214 outside the screening protocol). Of these, 73.2% were clinically localised and of low grade (T1-2NxM0 and Gleason <7). Similarly, of the 1489 FP men in the second round, 237 (15.9%) were in the following 4–7 years diagnosed with PC (77 at the third screen, 160 outside the screening protocol, 62.0% localised and of low grade). Of the 998 FP men in the third round, 26 (2.6%) developed PC later (in the following ⩽3 years, 38.5% were localised and of low grade).

## Discussion

Our results show that FP results affect every eighth man in repeated screening for PC with PSA even with a relatively high cutoff level of 4.0 ng ml^−1^. More than a quarter of the men with FP results are subsequently diagnosed with PC, although most of these cancers are localised and of low grade and have similar characteristics as cancers in men with a previous negative screening test. More than half of these men have persistent high serum PSA levels resulting in repeated FP results and biopsies. They are also at high risk of dropping out of subsequent screening.

The Finnish Prostate Cancer Screening Trial is part of the ERSPC study. There are some differences between the ERSPC centres in, for example, the mode of recruitment, screening interval, invitation procedures and the PSA threshold leading to biopsy. The Finnish trial is population-based and the largest of the ERSPC centres. A population-based study design ensures good generalisability at the population level.

The ERSPC study recently showed preliminary mortality results indicating a 20% relative decrease in mortality in the screening arm (Schröder *et al*, 2009). This was the first evidence for benefits from screening for PC with PSA. However, as shown by the ERSPC trial, 1410 men would have to be offered screening and 48 PCs treated to prevent one PC death during a 9-year period. In addition, the negative consequences of screening (adverse effects, including overdiagnosis, overtreatment and costs) still need to be carefully evaluated to allow assessment of the balance between benefits and harms before evidence-based decision-making concerning provision of screening can be made. This analysis contributes to that requirement.

Our study presents a similar proportion of FP results per screening episode as a previous Prostate, Lung, Colorectal and Ovarian (PLCO) Cancer Screening Trial study (Lafata *et al*, 2004), but also provides longer follow-up and information on the relation between FP results and several clinically important characteristics, such as PC, BPH medication, age and PSA level. Cumulative rates of FP results in repeated screening for several screening modalities were recently reported from the PLCO trial (Croswell *et al*, 2009). The authors showed a risk of 10.4% for at least one FP result in annual PSA screening during the 3-year screening period. We found a 12.5% risk for at least one FP result in two to three successive screening rounds during a 12-year follow-up, but the probability varied strongly with age. In the youngest age cohort (screened initially at age 55), the risk was 9.0% and in the oldest men (first screen at age 67 with only two rounds of screening) it was 15.7%. As previously noted, the incidence of PSA-elevating diseases other than PC (prostatitis, BPH) increases with age (Koskimäki *et al*, 1998; Rhodes *et al*, 1999; Wright *et al*, 2002) and explains the higher FP proportion in older men. This explanation is also consistent with the finding that men who used BPH medication had an increased risk for an FP result, despite the PSA-lowering effect of finasteride (Etzioni *et al*, 2005). These men were also older than men without BPH medication.

In our study, a PSA threshold of 4.0 ng ml^−1^ was used. In addition, during 1996–1998, men with a suspicious DRE finding and in 1999–2007 men with PSA 3.0–3.9 and free/total PSA ratio ⩽16% were referred. The PSA threshold was chosen in 1996 when the study began. A study from the Prostate Cancer Prevention Trial reported that 24.7% of men in the placebo group with PSA 2.1–4.0 had PC when biopsied at the end of the study, although 50% of these men were older than 71 years and all the cancers were stage T1 (Thompson *et al*, 2004). In another study with younger subjects (50–65 years), 11.3% (13 of 115) of men with PSA level 1.1–3.99 and F/T PSA ⩾20% had cancer in biopsy (Rowe *et al*, 2005). In our study with men aged 55–71 years, the proportion of PC from those biopsied varied from 26.6 to 32.9%. If the PSA threshold had been lower, for example, 2.1–3.9 ng ml^−1^, the proportion of PC in the biopsied men would probably be smaller, that is, the downside of the expected improvement in sensitivity would be a decreased specificity.

A screen-detected cancer was defined as a PC detected within 1 year from the PSA test in a man with a screen-positive result. On this basis, we defined an FP as a screen-positive result with no PC diagnosis within 1 year from the PSA test (excluding men without biopsy). Prostate-specific antigen predicts the development of PC by several years and there is no clear time as for the optimal definition of an FP result, but the proportion of *de novo* cases relative to those present at the screen can be anticipated to increase with time since the PSA test. If we had extended our 1-year limit to, for example, 3 years, the number of FP results would have decreased by 86 (6.5%), 88 (5.9%) and 47 (4.7%) men in the first, second and third rounds, respectively. These men were diagnosed with an interval cancer within 1–3 years from the PSA test. As the proportion of these men out of all FP men was relatively small (4.7–6.5%), using another definition would not be likely to materially affect our results.

In cancer screening, FP results are problematic for several reasons. Biopsies bring discomfort and often pain to the patient during the procedure (Mäkinen *et al*, 2002). Waiting for the result is a psychological strain, which can have negative effects for at least a year even after a negative biopsy result (Fowler *et al*, 2006). The economic impact of FP results has not been thoroughly analysed, but these men seem to receive more follow-up interventions such as PSA testing and re-biopsies, which add to the costs of screening (Lafata *et al*, 2004). Biopsy – similar to any invasive procedure – involves risks for adverse health effects, such as bleeding, infection or abscess formation (Mäkinen *et al*, 2002), although these complications are not very common.

There is previous evidence that FP men undergo more follow-up testing and biopsies than men with normal PSA (Fowler *et al*, 2006). Our results show that men with FP results receive more biopsies than do men who are diagnosed with PC. On average, every third FP man undergoes two biopsies within 4 years from the screen. It has been previously reported that the risk of clinically significant cancer decreases after the second biopsy (Djavan *et al*, 2003). Our study is likely to underestimate the average number of biopsies as we have no data on private sector visits and procedures and it is likely that some of the benign biopsies in the public sector are not reported to our database.

However, our findings indicate an increased risk for future PC with a history of one or several FP results. As many as 16% of FP men were diagnosed with PC in the next round. Most of the PCs were not aggressive, but, for example, in the third round as many as 29.9% of cancers were Gleason score 7 and 13% were advanced (T3-4NxM0 or TxNxM1 or Gleason score 8). Of the first round FP men, almost a third were diagnosed with PC during the 8–11 follow-up years. The proportion of PC diagnoses among the men with FP results at the second and third rounds were substantially lower (15.9 and 2.6%) – most likely because of a shorter follow-up. As previously mentioned, over 10% of men over 50 years of age can be diagnosed with PC even with low PSA levels (Rowe *et al*, 2005). Therefore, if men with an FP result receive more biopsies in the follow-up period than men with a negative screen, they could be more likely to receive a PC diagnosis because of more frequent biopsying. In addition, in 2002 we started using 10–12 core biopsies instead of sextant biopsies, which could increase the chances of finding small, indolent lesions during the later follow-up period. Both these factors increase the PC risk in FP men.

When the men were stratified by serum PSA level, it was evident that at the first (prevalence) screen, high PSA level was clearly associated with PC and moderately increased serum PSA level with FP. At the second and third (incidence) screens, these differences evened out and the positive predictive value of high PSA for PC decreased. The most likely explanation for these trends is that at the first screen most of the high PSA cancers were ‘harvested’ from the study population. Some of them were still detected at the second screen, but generally the cancers that produce high PSA were caught at the prevalence screen and few such cases arose *de novo* between the screening rounds.

In the PLCO trial, men with an FP result were almost twice more likely to decline subsequent screening compared with men with a negative screening result (Ford *et al*, 2005). Our results are similar, with RRs varying from 1.5 to 2.0. There might be several reasons behind this. The FP men could decide not to participate because of the unpleasant experience of unnecessary biopsy procedures and the anxiety related to the fear of PC diagnosis. On the other hand, an FP man could sense relief after a benign biopsy and deem it unnecessary to participate in the next screening round. Also, receiving a positive screening result without a confirmed PC diagnosis may erode a man's perception of the effectiveness of screening.

These findings emphasise the paradoxical problem of FP results in PC screening. On the one hand, FP men frequently have persistently high PSA levels (>50% chance of having another FP result in the next round) and undergo several biopsies. On the other hand, they are more likely to be diagnosed with PC, either because of biological processes or more active diagnostic procedures. New approaches are urgently needed for improved risk stratification among these men, that is, to predict which of them may harbour a clinically significant PC, which may have an insignificant indolent PC and which may have other factors underlying the elevated PSA level.

There is one weakness in our study. In some cases, the follow-up time after the third screen was relatively short (⩽3 years), as the last men were screened in the end of 2007 and follow-up ended in 2007. Therefore, some post-screening cancers were lacking for the last screening cohort. However, we believe that the strengths of this study well outweigh this weakness.

In conclusion, we present data from a prospective randomised controlled PC screening study spanning 12 years and three screening rounds. We have analysed the FP screening results during these rounds and calculated that every eighth man screened is subject to an FP result at least once in repeat screening. The men who receive FP results are likely to have a subsequent FP result(s) later if screened again. Also, these men commonly drop out of subsequent screening rounds. This poses a difficult equation, as men with FP results are at increased risk of being diagnosed later with a PC. More research is needed to balance the sensitivity and specificity of PC screening to minimise the proportion of FP results.

## Figures and Tables

**Table 1:** Screening results by age and screening round

	**Screen negative (%)**	**False positive (%)**	**Prostate cancer (%)**	**Not biopsied (%)**	**Total**
*Round I*					
55 years	6146 (95.2)	210 (3.3)	76 (1.2)	21 (0.3)	6453
59 years	5136 (92.5)	274 (4.9)	130 (2.3)	12 (0.2)	5552
63 years	4143 (87.6)	397 (8.4)	153 (3.2)	35 (0.7)	4728
67 years	3387 (83.5)	450 (11.1)	184 (4.5)	35 (0.9)	4056
Total	18 812 (90.5)	1331 (6.4)	543 (2.6)	103 (0.5)	20 789
					
*Round II*					
59 years	5700 (92.4)	313 (5.1)	115 (1.9)	39 (0.6)	6167
63 years	4464 (88.0)	401 (7.9)	158 (3.1)	48 (0.9)	5071
67 years	3426 (84.9)	372 (9.2)	181 (4.5)	58 (1.4)	4037
71 years	2719 (81.5)	403 (12.1)	159 (4.8)	57 (1.7)	3338
Total	16 309 (87.6)	1489 (8.0)	613 (3.3)	202 (1.1)	18 613
					
*Round III*					
63 years	4833 (89.5)	352 (6.5)	157 (2.9)	59 (1.1)	5401
67 years	3618 (86.5)	334 (8.0)	164 (3.9)	66 (1.6)	4182
71 years	2645 (83.8)	312 (9.9)	134 (4.2)	66 (2.1)	3157
Total	11 096 (87.1)	998 (7.8)	455 (3.6)	191 (1.5)	12 740
					
Total	46 217 (88.6)	3818 (7.3)	1611 (3.1)	496 (1.0)	52 142

Non-participants omitted.

**Table 2:** Risks for next-round prostate cancer, FP result and non-participation after a previous round FP result and negative screening result

	**Risk for PC after FP result, %**	**Risk for PC after negative screen, %**	**RR (95 % CI)**	**Risk for FP result after FP result, %**	**Risk for FP after negative screen, %**	**RR (95 % CI)**	**Risk for non- participation after FP result, %**	**Risk for non-participation after negative screen, %**	**RR (95 % CI)**
*Round 1/Round 2*									
55 years	14.1	1.4	9.9 (6.1–16.1)	50.0	4.1	12.2 (9.8–15.1)	27.8	16.4	1.7 (1.3–2.2)
59 years	15.2	2.4	6.3 (4.3–9.4)	64.0	5.5	11.6 (9.8–13.7)	21.6	14.3	1.5 (1.2–1.9)
63 years	19.7	3.2	6.2 (4.5–8.5)	53.6	6.0	9.0 (7.5–10.7)	26.8	14.5	1.8 (1.5–2.2)
67 years	14.2	3.7	3.9 (2.7–5.5)	62.5	7.1	8.8 (7.4–10.4)	27.3	14.0	1.9 (1.6–2.3)
Total	16.0	2.5	6.5 (5.4–7.8)	58.3	5.4	10.7 (9.8–11.7)	26.0	15.0	1.7 (1.6–1.9)
									
*Round 2/Round 3*									
59 years	12.9	2.1	6.2 (4.0–9.6)	52.0	4.3	12.0 (9.8–14.6)	29.3	16.7	1.8 (1.4–2.1)
63 years	12.3	3.4	3.6 (2.4–5.3)	59.1	4.5	13.3 (11.0–16.0)	29.6	14.6	2.0 (1.7–2.4)
67 years	13.6	3.0	4.5 (3.0–6.7)	58.7	5.9	10.0 (8.3–12.1)	29.3	16.3	1.8 (1.5–2.2)
Total	12.9	2.8	4.7 (3.7–5.9)	57.0	4.8	12.0 (10.7–13.4)	29.4	15.9	1.8 (1.7–2.1)

Abbreviations: PC=prostate cancer; FP=false-positive screening result; RR=risk ratio; CI=confidence interval.

**Table 3:** Proportions of men with false-positive results and prostate cancer by serum PSA concentration in the three rounds of the trial

	**Men, *N* (%)**	**Men biopsied, *N* (%)**	**False positives, *N* (%)**	**Prostate cancer, *N* (%)**
*Round 1*				
* PSA* (*ng ml*^−*1*^)				
⩽2.9 or 3.0–3.9 and aux. test −	18 812 (90.5)	—	—	—
3.0–3.9 and aux. test +	149 (0.7)	142 (95.3)	105 (73.9)	37 (26.1)
4.0–9.9	1527 (7.3)	1440 (94.3)	1110 (77.1)	330 (22.9)
⩾10.0	301 (1.4)	292 (97.0)	116 (39.7)	176 (60.3)
Total	20 789	1874 (9.0)	1331 (6.4)	543 (2.6)
				
*Round 2*				
* PSA* (*ng ml*^−*1*^)				
⩽2.9 or 3.0–3.9 and aux. test –	16 309 (87.6)	—	—	—
3.0–3.9 and aux. test +	232 (1.2)	215 (92.7)	147 (68.4)	68 (31.6)
4.0–9.9	1819 (9.8)	1653 (90.9)	1203 (72.8)	450 (27.2)
⩾10.0	253 (1.4)	234 (92.5)	139 (59.4)	95 (40.6)
Total	18 613	2102 (11.3)	1489 (8.0)	613 (3.3)
				
*Round 3*				
* PSA* (*ng ml*^−*1*^)				
⩽2.9 or 3.0–3.9 and aux. test –	11 096 (87.1)	—	—	—
3.0–3.9 and aux. test +	160 (1.3)	142 (88.8)	88 (62.0)	54 (38.0)
4.0–9.9	1328 (10.4)	1167 (87.9)	813 (69.7)	354 (30.3)
⩾10.0	156 (1.2)	144 (92.3)	97 (67.4)	47 (32.6)
Total	12 740	1453 (11.4)	998 (7.8)	455 (3.6)

Abbreviations: PSA=prostate-specific antigen; aux. test=auxiliary test.

Auxiliary test: 1996–1998 digital rectal examination, 1999–2007 free/total PSA ratio (cutoff 16%).
